# D-dimer after 3 months of anticoagulation therapy and outcomes in cancer-associated isolated distal deep vein thrombosis

**DOI:** 10.1016/j.bvth.2025.100063

**Published:** 2025-02-24

**Authors:** Tatsuya Nishikawa, Yugo Yamashita, Masashi Fujita, Takeshi Morimoto, Nao Muraoka, Michihisa Umetsu, Yuji Nishimoto, Takuma Takada, Yoshito Ogihara, Nobutaka Ikeda, Kazunori Otsui, Daisuke Sueta, Yukari Tsubata, Masaaki Shoji, Ayumi Shikama, Yutaka Hosoi, Yasuhiro Tanabe, Ryuki Chatani, Kengo Tsukahara, Naohiko Nakanishi, Kitae Kim, Satoshi Ikeda, Taku Yasui, Hironori Yamamoto, Koh Ono, Takeshi Kimura, ONCO DVT Study Investigators

**Affiliations:** 1 Department of Onco-Cardiology, Osaka International Cancer Institute, Osaka, Japan; 2 Department of Cardiology, Akashi Medical Center, Akashi, Japan; 3 Department of Cardiovascular Medicine, Graduate School of Medicine, Kyoto University, Kyoto, Japan; 4 Department of Clinical Epidemiology, Hyogo Medical University, Nishinomiya, Japan; 5 Division of Cardiology, Shizuoka Cancer Center, Nagaizumi, Japan; 6 Division of Vascular Surgery, Department of Surgery, Tohoku University Hospital, Sendai, Japan; 7 Division of Cardiology, Osaka General Medical Center, Osaka, Japan; 8 Department of Cardiology, Tokyo Women’s Medical University, Tokyo, Japan; 9 Department of Cardiology and Nephrology, Mie University Graduate School of Medicine, Tsu, Japan; 10 Division of Cardiovascular Medicine, Toho University Ohashi Medical Center, Tokyo, Japan; 11 Department of General Internal Medicine, Kobe University Graduate School of Medicine, Kobe, Japan; 12 Department of Cardiovascular Medicine, Graduate School of Medical Sciences, Kumamoto University, Kumamoto, Japan; 13 Division of Medical Oncology and Respiratory Medicine, Department of Internal Medicine, Shimane University Faculty of Medicine, Izumo, Japan; 14 Department of Cardiovascular Medicine, National Cancer Center Hospital, Tokyo, Japan; 15 Department of Obstetrics and Gynecology, Faculty of Medicine, University of Tsukuba, Tsukuba, Japan; 16 Department of Cardiovascular Surgery, Kyorin University Faculty of Medicine, Tokyo, Japan; 17 Department of Cardiology, St. Marianna University School of Medicine, Kawasaki, Japan; 18 Department of Cardiovascular Medicine, Kurashiki Central Hospital, Kurashiki, Japan; 19 Division of Cardiology, Fujisawa City Hospital, Fujisawa, Japan; 20 Department of Cardiovascular Medicine, Graduate School of Medical Science, Kyoto Prefectural University of Medicine, Kyoto, Japan; 21 Department of Cardiovascular Medicine, Kobe City Medical Center General Hospital, Kobe, Japan; 22 Department of Cardiovascular Medicine, Nagasaki University Graduate School of Biomedical Sciences, Nagasaki, Japan; 23 Department of Cardiology, Hirakata Kohsai Hospital, Hirakata, Japan

## Abstract

Cancer-associated isolated distal deep vein thrombosis (IDDVT) is a common complication in patients with cancer. The Optimal Duration of Anticoagulation Therapy for Isolated Distal Deep Vein Thrombosis in Patients with Cancer study revealed the superiority of 12- over 3-month edoxaban treatment with respect to thrombotic risk. However, it remains unclear whether D-dimer levels during anticoagulation influence the efficacy of extended anticoagulation. In this post hoc subgroup analysis, we stratified 519 patients into the low D-dimer (<1.0 μg/mL) (n = 308) and high D-dimer (≥1.0 μg/mL) (n = 211) subgroups based on D-dimer levels at 3 months. The cumulative incidence of a composite of symptomatic recurrent venous thromboembolism (VTE) or VTE-related death was lower in the 12-month edoxaban group than in the 3-month edoxaban group in both the low D-dimer (0.8% vs 5.6%; *P* = .02; odds ratio [OR], 0.12; 95% confidence interval [CI], 0.01-0.66) and high D-dimer (0.9% vs 10.2%; *P* = .01; OR, 0.11; 95% CI, 0.01-0.62) subgroups without interaction. Furthermore, there was no significant difference in the cumulative incidence of major bleeding between the 12- and 3-month groups in both the low D-dimer (3.6% vs 1.8%; *P* = .64; OR, 1.96; 95% CI, 0.47-9.67) and high D-dimer (18.3% vs 14.6%; *P* = .29; OR, 1.27; 95% CI, 0.60-2.75) subgroups without interaction. In conclusion, a 12-month edoxaban treatment for cancer-associated IDDVT was superior to a 3-month treatment in reducing thrombotic events, irrespective of D-dimer levels after 3 months of anticoagulation therapy. There was no significant increased risk of major bleeding in the 12-month edoxaban group relative to the 3-month edoxaban group regardless of the D-dimer levels at 3 months. This trial was registered at www.clinicaltrials.gov as #NCT03895502.

## Key Points

• Compared with a 3-month treatment, a 12-month edoxaban treatment is superior in reducing thrombotic events regardless of the D-dimer levels.
• A 12-month edoxaban treatment does not significantly increase the risk of major bleeding regardless of the D-dimer levels.

## Visual Abstract

**Figure:**
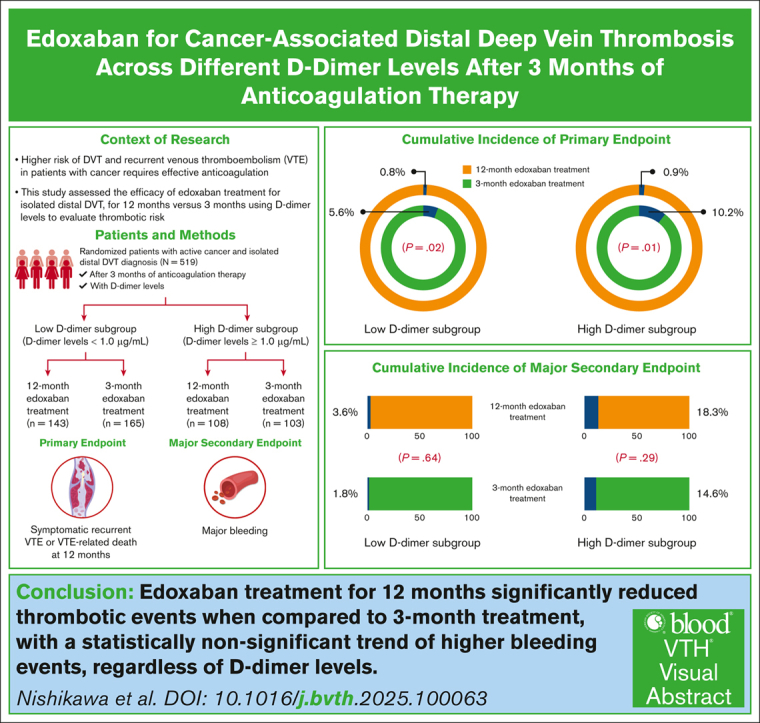

## Introduction

Cancer-associated venous thromboembolism (VTE), with higher risks of recurrent VTE, bleeding events, and mortality compared with VTE without cancer, is 1 of the major concerns related to prognosis and quality of life in patients with cancer.^1^ Thus, appropriate diagnosis and treatment for cancer-associated VTE are important in daily clinical practice. D-dimer levels have often been evaluated as a biomarker of thrombosis during VTE management, and D-dimer levels at diagnosis have historically been used for the diagnosis of VTE. In particular, several prediction scores for VTE development include D-dimer levels at diagnosis as a score component.^2^, ^3^, ^4^ In addition, D-dimer levels can be useful for the risk stratification of prognosis, including in the prediction of recurrent VTE. Several studies reported that D-dimer levels during VTE treatment are useful for predicting recurrent VTE.^5^, ^6^, ^7^, ^8^, ^9^, ^10^ However, there have been limited data on the utility of D-dimer levels during VTE treatment for the risk stratification of recurrent VTE in patients with cancer-associated VTE. Recently, a randomized clinical trial, the Optimal Duration of Anticoagulation Therapy for Isolated Distal Deep Vein Thrombosis in Patients with Cancer (ONCO DVT) study, revealed that 12-month edoxaban treatment was associated with a lower risk of recurrent VTE than 3-month edoxaban treatment in patients with cancer and isolated distal deep vein thrombosis (DVT).^11^ However, the risk of recurrent VTE can vary widely among patients, and further risk assessment, including D-dimer levels during the treatment course, can be useful for selecting patients who can benefit more from 12-month edoxaban treatment. Therefore, this post hoc analysis of the ONCO DVT study aimed to evaluate the effect of D-dimer levels after 3 months of anticoagulant therapy on clinical outcomes.

## Methods

### Study design

This study was a post hoc analysis of the ONCO DVT study (ClinicalTrials.gov identifier: NCT03895502), which was an investigator-initiated, multicenter, open-label, adjudicator-blinded, superiority randomized clinical trial conducted at 60 institutions across Japan (supplemental Appendix 1). Full details of the ONCO DVT study have been described previously.^11^ In brief, patients with active cancer who were newly diagnosed with isolated distal DVT using ultrasonography were randomly assigned in a 1:1 ratio to receive either 12- or 3-month edoxaban treatment. At the 3-month visit after the diagnosis (between 61 and 119 days), the 3-month edoxaban treatment group stopped edoxaban, whereas the 12-month edoxaban treatment group continued edoxaban. The trial was conducted in line with the principles of the Declaration of Helsinki and was approved by the Kyoto University Institutional Review Board and the institutional review boards of all participating institutions (supplemental Appendix 2). Written informed consent was obtained from all study participants.

### Study population

**Figure 1.**
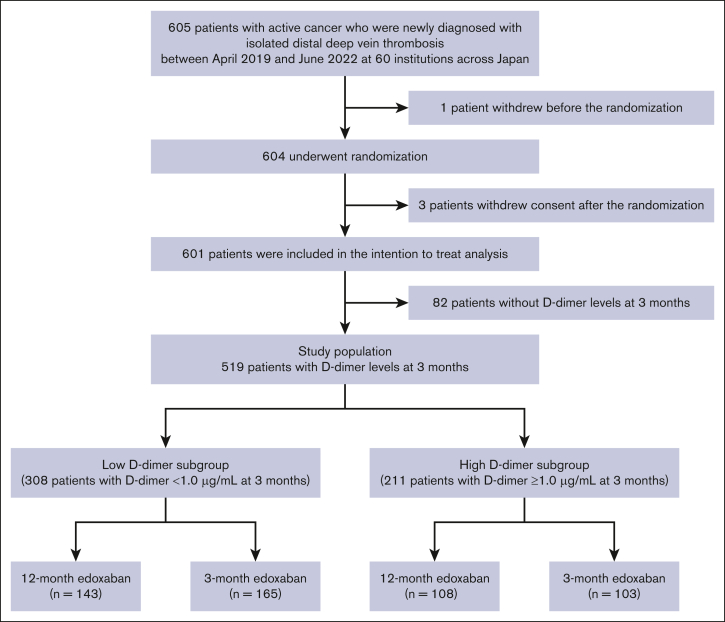
**Study flowchart.**

A total of 604 patients were randomized from April 2019 to June 2022. After excluding 3 patients who withdrew consent after randomization and 82 patients without data on D-dimer levels at 3 months, 519 patients were finally included in the current study (Figure 1). We divided the study population into 2 subgroups according to D-dimer levels after 3 months of edoxaban treatment with a cutoff value of 1.0 μg/mL: patients with D-dimer levels <1.0 μg/mL (low D-dimer subgroup) and those with D-dimer levels ≥1.0 μg/mL (high D-dimer subgroup). The cutoff value of 1.0 μg/mL was determined on the basis of previous reports in the field of VTE.^12^^,^^13^ Although the cutoff value of 0.5 μg/mL or the age-adjusted value for older adult patients has also often been used to diagnose or exclude VTE, limited data are available on its validation in patients with cancer-associated VTE. Given the typically high D-dimer levels in patients with cancer, a cutoff value of 1.0 μg/mL was selected arbitrarily in the present study. D-dimer levels were evaluated at the 3-month visit (between 61 and 119 days) at each institution, which had been predefined in the study protocol.

### Definitions of patient characteristics

Active cancer was defined as the presence of 1 of the following: (1) newly diagnosed cancer within 6 months of randomization; (2) cancer treatment, including surgery, chemotherapy, and radiotherapy, performed within 6 months of randomization; (3) currently receiving cancer treatment, including surgery, chemotherapy, or radiotherapy; (4) recurrence, local invasion, or distant metastases; and (5) hematopoietic malignancies without complete remission. Definitions of other baseline characteristics are provided in supplemental Appendix 3.

### Definitions of clinical end points

The primary and major secondary end points in the current study were identical to those in the primary analysis of the ONCO DVT study. The primary end point was a composite of symptomatic recurrent VTE or VTE-related death at 12 months. Symptomatic recurrent VTE was defined as new thrombi on imaging tests or worsening thrombi compared with the most recent imaging, with new or recently worsening pulmonary embolism (PE) or DVT symptoms. VTE-related death was defined as objectively confirmed PE before death or autopsy-confirmed PE, without another more likely cause of death, or death most likely related to PE despite not being objectively confirmed. The major secondary end point was major bleeding events at 12 months. Major bleeding was defined as fatal bleeding, symptomatic bleeding in a critical area or organ, and bleeding reducing hemoglobin levels by ≥2 g/dL or requiring transfusions of ≥2 units of whole blood or red cells according to the International Society on Thrombosis and Haemostasis criteria.^14^

Other secondary end points were asymptomatic recurrent VTE, all clinically relevant bleeding, and all-cause death. The definitions of these end points are shown in supplemental Appendix 4. Persistent edoxaban discontinuation was defined as a discontinuation of edoxaban according to the study protocol or lasting for >14 days for any reason. The members of an independent clinical events committee who were unaware of the study group assignments adjudicated all suspected outcome events and causes of death, as well as the severity of the major bleeding events, using prespecified criteria.

### Statistical analysis

Categorical variables are presented as numbers and percentages, whereas continuous variables are presented as mean and standard deviation or median and interquartile range (IQR) depending on their distributions. Categorical variables were compared using the chi-squared test or Fisher exact test, and continuous variables were compared using the Student *t* test or Wilcoxon rank-sum test according to their distributions. The cumulative incidence was estimated using the Kaplan-Meier method, and the differences between the 12- and 3-month edoxaban groups were assessed using the log-rank test. The index date for the Kaplan-Meier method was the time of diagnosis of isolated distal DVT. We calculated odds ratios (OR) with the corresponding 95% confidence intervals (CI) using logistic regression models, and differences in the effects of 12- and 3-month edoxaban treatment were evaluated between the subgroups using interaction terms in the models. In addition, to evaluate clinical outcomes beyond 3 months, we conducted a landmark analysis at 90 days. In this analysis, we excluded patients who died within 90 days and those who experienced the clinical end point. Furthermore, to account for the competing risk of all-cause death, we used the Fine-Gray method for the primary and major secondary end points. We also conducted a sensitivity analysis using a different cutoff value of 0.5 μg/mL instead of 1.0 μg/mL. The reported *P* values were 2-tailed, and statistical significance was set at *P* < .05. JMP version 11.0 (SAS Institute Inc, Cary, NC) was used for all analyses, except the Fine-Gray model analysis, which was conducted using EZR version 1.68 (https://www.jichi.ac.jp/saitama-sct/SaitamaHP.files/statmedEN.html). The Kaplan-Meier curves were generated using GraphPad Prism 9 (GraphPad Software Inc, San Diego, CA).

## Results

### Patient characteristics

Among the 519 patients included in the current study, 308 (59%) had D-dimer levels <1.0 μg/mL (low D-dimer subgroup) and 211 (41%) had D-dimer levels ≥1.0 μg/mL (high D-dimer subgroup) at 3 months (Figure 1). The median interval between randomization and measurement of D-dimer level at the 3-month visit was 92 (IQR, 84-104) days for the low D-dimer subgroup and 88 (IQR, 72-97) days for the high D-dimer subgroup. There was no significant difference in mean age (69.7 vs 71.0 years; *P* = .17) and body weight (56.5 vs 55.1 kg; *P* = .20) between the subgroups (Table 1). Regarding cancer status, the prevalence of metastasis (18% vs 30%; *P* < .001) and performance status ≥2 (9% vs 22%; *P* < .001) was significantly lower in the low D-dimer subgroup than in the high D-dimer subgroup. The D-dimer level at randomization was also lower in the low D-dimer subgroup than in the high D-dimer subgroup (3.9 [IQR, 1.8-8.8] vs 7.1 [IQR, 3.1-14.4] μg/mL; *P* < .001). The median D-dimer level at the 3-month visit was 0.5 (IQR, 0.5-0.7) for the low D-dimer subgroup and 2.5 (IQR, 1.5-5.5) for the high D-dimer subgroup. The clinical characteristics of the patients at baseline were well balanced between the 12- and 3-month edoxaban groups in both subgroups (supplemental Table 1). Details of the cancer types are presented in supplemental Table 2.

**Table 1.** **Patient characteristics**

	Low D-dimer subgroup (D-dimer <1.0 μg/mL at 3 months) n = 308	High D-dimer subgroup (D-dimer ≥1.0 μg/mL at 3 months) n = 211	*P* value
**Baseline characteristics**			
Age, y	69.7 ± 10.1	71.0 ± 9.8	.17
Age ≥75 y, n (%)	115 (37)	83 (39)	.65
Male sex, n (%)	70 (23)	68 (32)	.02
Body weight, kg	56.5 ± 13.0	55.1 ± 10.8	.20
Body mass index, kg/m^2^	23.0 ± 4.4	22.3 ± 3.8	.052
Site of DVT			
Bilateral sides, n (%)	103 (33)	86 (41)	.13
Right side, n (%)	90 (29)	47 (22)	
Left side, n (%)	115 (37)	78 (37)	
Standard dose of edoxaban (60 mg per day)∗	83 (27)	53 (25)	.69
Lower dose of edoxaban (30 mg per day)∗	225 (73)	158 (75)	
**Cancer status, n (%)**			
Newly diagnosed cancer within 6 months	207 (67)	133 (63)	.35
Chemotherapy performed within 6 months	144 (47)	113 (54)	.13
Radiotherapy performed within 6 months	22 (7)	23 (11)	.15
Recurrent cancer	34 (11)	17 (8)	.30
Metastatic disease	54 (18)	64 (30)	<.001
ECOG performance status			
0	185 (60)	96 (46)	<.001
1	95 (31)	68 (32)	
≥2	28 (9)	47 (22)	
**Comorbidities, n (%)**			
Hypertension	123 (40)	97 (46)	.18
Diabetes	44 (14)	40 (19)	.18
Heart failure	2 (1)	3 (1)	.33
History of stroke	14 (5)	9 (4)	1.00
History of VTE	14 (5)	15 (7)	.25
History of major bleeding	11 (4)	9 (4)	.82
Transient risk factors for VTE	75 (24)	50 (24)	.92
**Laboratory values at diagnosis**			
Creatinine clearance ≤50 mL/min, n (%)	57 (19)	46 (22)	.37
Anemia†, n (%)	183 (59)	158 (75)	<.001
Platelet count, ×10^3^/μL	250 ± 111	255 ± 119	.66
D-dimer level at randomization, μg/mL	3.9 (1.8-8.8)	7.1 (3.1-14.4)	<.001
D-dimer level at 3 months, μg/mL	0.5 (0.5-0.7)	2.5 (1.5-5.5)	<.001
**Concomitant medication, n (%)**			
Antiplatelet	23 (7)	12 (6)	.48
Steroid	30 (10)	36 (17)	.02
Statin	70 (23)	40 (19)	.30
Proton pump inhibitor	89 (29)	80 (38)	.04
H_2_ blocker	19 (6)	10 (5)	.56

Continuous variables are presented as median (IQR) or mean ± standard deviation. Categorical variables are presented as number (%).

ECOG, Eastern Cooperative Oncology Group; H_2_, histamine.

∗ Edoxaban is administered at a lower dose of 30 mg/d for the participants with a creatinine clearance of 30 to 50 mL/min, body weight of ≤60 kg, or concomitant treatment with potent P-glycoprotein inhibitors.

† Anemia is defined as a hemoglobin level of <13 g/dL for men or <12 g/dL for women.

### Edoxaban treatment

**Figure 2.**
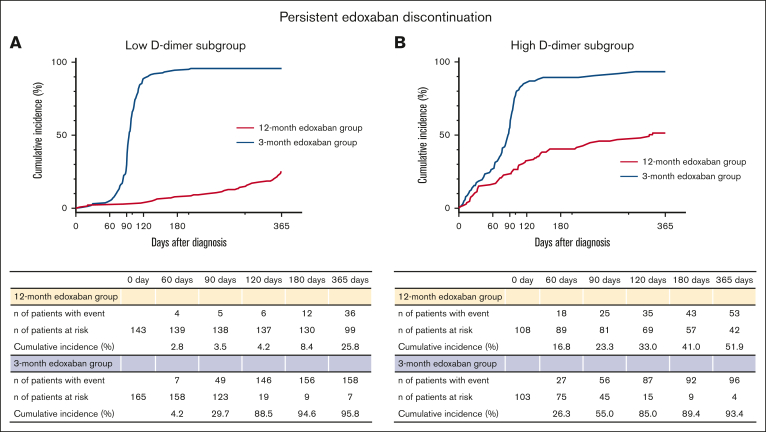
**Kaplan-Meier curves for persistent edoxaban discontinuation comparing the 12- and 3-month edoxaban groups in the subgroups stratified by D-dimer levels at 3 months.** (A) Low D-dimer subgroup. (B) High D-dimer subgroup. Time-to-event curves for persistent edoxaban discontinuation. Persistent edoxaban discontinuation is defined as the discontinuation of edoxaban according to the study protocol or lasting >14 days for any reason.

In the low D-dimer subgroup, the cumulative 120-day incidence of persistent edoxaban discontinuation was 4.2% in the 12-month edoxaban group and 88.5% in the 3-month edoxaban group, with a median duration of edoxaban treatment of 365 and 95 days, respectively (Figure 2A). In the high D-dimer subgroup, the cumulative 120-day incidence of persistent edoxaban discontinuation was 33.0% in the 12-month edoxaban group and 85.0% in the 3-month edoxaban group, with a median duration of edoxaban treatment of 214 and 87 days, respectively (Figure 2B). The details of the reasons for persistent edoxaban discontinuation are presented in supplemental Table 3. In the low D-dimer subgroup, the primary reasons for persistent edoxaban discontinuation were bleeding events (25%) in the 12-month edoxaban group and per-protocol discontinuation (93%) in the 3-month edoxaban group. In the high D-dimer subgroup, the reasons were cancer progression (30%) in the 12-month edoxaban group and per-protocol discontinuation (62%) in the 3-month edoxaban group.

### Primary end points

**Figure 3.**
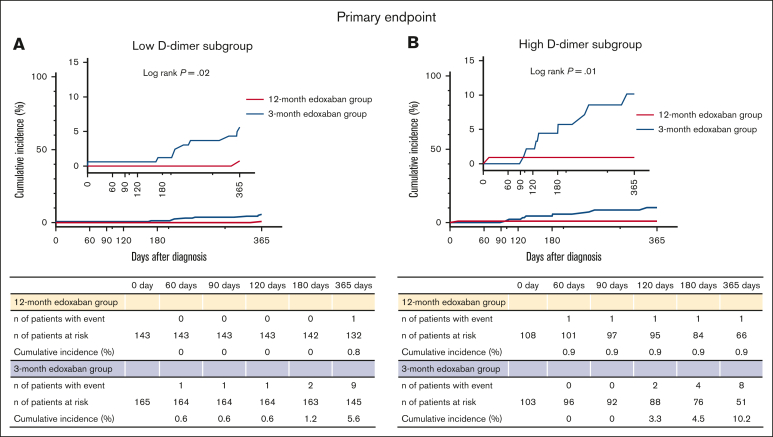
**Kaplan-Meier curves for the primary end point comparing the 12- and 3-month edoxaban groups in the subgroups stratified by D-dimer levels at 3 months.** (A) Low D-dimer subgroup. (B) High D-dimer subgroup. The primary end point is a composite of symptomatic recurrent VTE or VTE-related death.

**Figure 4.**
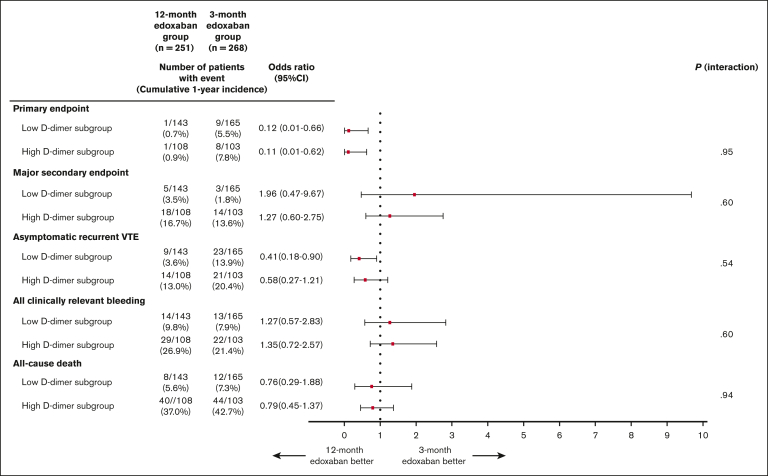
**Clinical end points comparing the 12- and 3-month edoxaban groups in the subgroups stratified by D-dimer levels at 3 months.** The primary end point is a composite of symptomatic recurrent VTE or VTE-related death. The major secondary end point is major bleeding as defined according to the International Society on Thrombosis and Haemostasis criteria, and consists of fatal bleeding, symptomatic bleeding in a critical area or organ, and bleeding causing a reduction in hemoglobin levels by ≥2 g/dL or leading to a transfusion of ≥2 units of whole blood or red cells.

The cumulative incidence of the primary end point was lower in the 12-month edoxaban group than in the 3-month edoxaban group in both the low D-dimer subgroup (0.8% vs 5.6%; log-rank *P* = .02; OR, 0.12; 95% CI, 0.01-0.66) (Figure 3A) and high D-dimer subgroup (0.9% vs 10.2%; log-rank *P* = .01; OR, 0.11; 95% CI, 0.01-0.62) (Figure 3B). There was no significant interaction between the subgroups for the primary end point (*P* = .95) (Figure 4). Furthermore, the competing risk analysis, which considered death a competing risk, showed consistent results for both the low D-dimer subgroup (Gray test, *P* = .02) and high D-dimer subgroup (Gray test, *P* = .01). The cumulative incidence of the primary end point in the 3-month edoxaban group was higher in the high D-dimer subgroup than in the low D-dimer subgroup (10.2% vs 5.6% at 365 days), whereas the cumulative incidence of the primary end point in the 12-month edoxaban group was similar across the 2 subgroups (0.9% vs 0.8% at 365 days) (Figure 3).

### Major secondary end points

**Figure 5.**
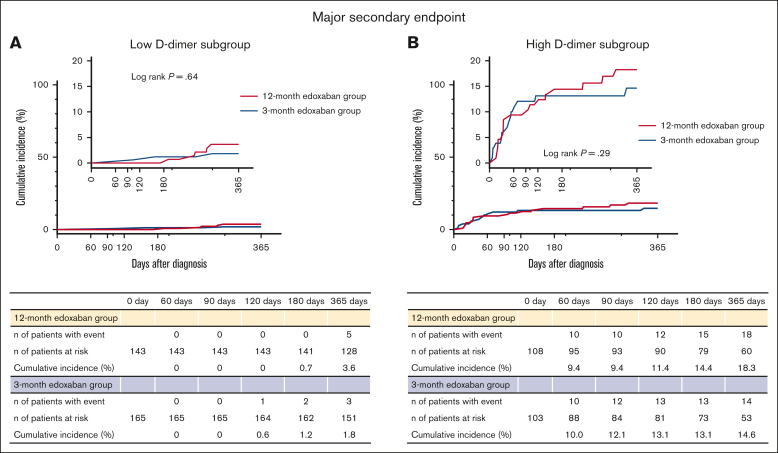
**Kaplan-Meier curves for the major secondary end point comparing the 12- and 3-month edoxaban groups in the subgroups stratified by D-dimer levels at 3 months.** (A) Low D-dimer subgroup. (B) High D-dimer subgroup. The major secondary end point is major bleeding as defined according to the International Society on Thrombosis and Haemostasis criteria, and consists of fatal bleeding, symptomatic bleeding in a critical area or organ, and bleeding causing a reduction in hemoglobin levels by ≥2 g/dL or leading to a transfusion of ≥2 units of whole blood or red cells.

There was no significant difference in the cumulative incidence of the major secondary end point between the 12- and 3-month edoxaban groups in both the low D-dimer subgroup (3.6% vs 1.8%; log-rank *P* = .64; OR, 1.96; 95% CI, 0.47-9.67) (Figure 5A) and the high D-dimer subgroup (18.3% vs 14.6%; log-rank *P* = .29; OR, 1.27; 95% CI, 0.60-2.75) (Figure 5B), without interaction between the subgroups and the lower risk of 12-month edoxaban treatment relative to 3-month edoxaban treatment for the major secondary end point (*P* = .60) (Figure 4). Furthermore, the competing risk analysis regarding death as a competing risk showed consistent results for both the low D-dimer subgroup (Gray test, *P* = .35) and the high D-dimer subgroup (Gray test, *P* = .57). In the 3-month edoxaban group, the cumulative incidence of the major secondary end point was higher in the high D-dimer subgroup than in the low D-dimer subgroup (14.6% vs 1.8% at 365 days). Similarly, in the 12-month edoxaban group, the cumulative incidence of the major secondary end point was also higher in the high D-dimer subgroup than in the low D-dimer subgroup (18.3% vs 3.6% at 365 days) (Figure 5).

### Other secondary end points, landmark analysis, and sensitivity analysis

The results of the other secondary end points are described in Figure 4 and supplemental Figures 1-3. There was no significant difference in the cumulative incidence of all-cause death between the 12- and 3-month edoxaban groups in the low and high D-dimer subgroups (5.7% vs 7.3%; log-rank *P* = .57; 37.4% vs 43.5%; log-rank *P* = .40) (supplemental Figure 3A-B). The cumulative incidences of all-cause death in both the 12- and 3-month edoxaban groups were higher in the high D-dimer subgroup than in the low D-dimer subgroup.

In the landmark analysis at 90 days, the cumulative incidence of the primary end point was significantly lower in the 12-month edoxaban group than in the 3-month edoxaban group in both the low and high D-dimer subgroups (0.8% vs 5.1%; log-rank *P* = .03; 0% vs 10.2%; log-rank *P* = .003) (supplemental Figure 4A-B). Furthermore, there was no significant difference in the cumulative incidence of the major secondary end point between the 12- and 3-month edoxaban groups in both the low and high D-dimer subgroups (3.6% vs 1.8%; log-rank *P* = .35; 9.8% vs 2.9%; log-rank *P* = .08) (supplemental Figure 5A-B). The sensitivity analysis using a different cutoff value of 0.5 μg/mL showed a trend similar to that observed in the main analysis (supplemental Figures 6 and 7).

## Discussion

The main findings of the current study were as follows. First, 12-month edoxaban treatment was superior to 3-month edoxaban treatment with respect to thrombotic risk, regardless of the D-dimer levels after 3 months of anticoagulation therapy. Second, there was no significant increase in the risk of major bleeding in the 12-month edoxaban group relative to the 3-month edoxaban group, regardless of the D-dimer levels after 3 months of anticoagulation therapy.

These results were consistent with the primary report of the ONCO DVT study,^11^ irrespective of the D-dimer levels after 3 months of anticoagulation therapy. The current results also provided several insights into the utility of D-dimer levels after 3 months of anticoagulation therapy. A previous study reported a higher incidence of recurrent VTE in patients with abnormal D-dimer levels 1 month after discontinuation of anticoagulation than in those with normal levels, and the incidence was reduced by the resumption of anticoagulation therapy.^6^ Other studies also reported that abnormal D-dimer levels during the course of anticoagulation treatment for VTE were associated with a higher thrombotic risk than normal D-dimer levels, suggesting the utility of D-dimer levels after anticoagulation therapy for decision-making regarding anticoagulation strategies for these patients.^8^^,^^15^

However, these previous studies did not include patients with active cancer, and it remains unknown whether this approach can be applied to patients with cancer-associated VTE. Our data from the ONCO DVT study suggested that the continuation of edoxaban treatment beyond 3 months was superior to the discontinuation of edoxaban treatment at 3 months with respect to the thrombotic risk not only among patients with D-dimer levels above the cutoff at 3 months but also among those with D-dimer levels below the cutoff. These results might suggest that the thrombotic risk of cancer-associated isolated distal DVT is not low even among patients with D-dimer levels below the cutoff after anticoagulation therapy. Further, the current study also revealed that the crude cumulative incidence rate of recurrent VTE in the 3-month edoxaban group was lower among patients with D-dimer levels below the cutoff at 3 months than among those with D-dimer levels above the cutoff (5.6% vs 10.2%). Patients with D-dimer levels below the cutoff had a lower prevalence of metastatic cancer and performance status ≥2 compared with those with D-dimer levels above the cutoff. These results suggest that patients with D-dimer levels above the cutoff at 3 months might have a high risk of recurrent VTE due to the effect of advanced cancer, and deserve prolonged anticoagulation therapy.

Notably, the current study showed that the incidence rate of persistent edoxaban discontinuation was generally higher among patients with D-dimer levels above the cutoff at 3 months. In addition to the high incidence rate of discontinuation even in the 12-month edoxaban treatment group, the rate of early discontinuation appeared to be high among patients with D-dimer levels above the cutoff at 3 months. This could be partly due to a generally higher risk of major bleeding among patients with D-dimer levels above the cutoff at 3 months than among those with D-dimer levels below the cutoff. In the current study, those with D-dimer levels above the cutoff had more advanced cancer, which might explain the higher incidence of major bleeding compared with those with D-dimer levels below the cutoff. The abnormal D-dimer levels, even after anticoagulation therapy, can reflect an abnormality in the fibrinolytic system and high cancer activity, which might lead to a higher risk of bleeding events. Given the trend of a higher risk of major bleeding beyond 3 months among patients with D-dimer levels above the cutoff compared with those below the cutoff in the 12-month edoxaban treatment group, clinicians should exercise caution in balancing thrombotic and bleeding risks for these patients. Considering the wide variety of patients with cancer, further studies are warranted to stratify the detailed thrombotic and bleeding risks per patient, including individualized management.

The current study has several limitations. First, the open-label design had the potential to introduce bias, such as ascertainment bias. However, all clinical end points were adjudicated by the members of the independent committee who were blinded to the study group assignments. Second, some patients had missing D-dimer level values at the 3-month visit, leading to potential selection bias, although the evaluation of D-dimer levels at the 3-month visit had been predefined in the study protocol. In addition, we evaluated D-dimer levels at 3 months, regardless of anticoagulation status at that time. Thus, D-dimer values were assessed during anticoagulation therapy in some patients, but not in others. Third, there may be racial differences, and the generalizability of the current results should be carefully considered, including their application to non-Japanese populations. Fourth, there is no established cutoff value for D-dimer, and we could not determine the optimal cutoff value in this study. Moreover, although a contemporary latex agglutination assay was used in all participating institutions, D-dimer levels were assessed at each institution according to local protocols, which could cause variations in D-dimer assessment. Furthermore, we did not evaluate the exact timing of edoxaban intake relative to the blood test, nor could we discuss D-dimer values in relation to peak or trough edoxaban levels.

In conclusion, a 12-month edoxaban treatment for cancer-associated isolated distal DVT was superior to a 3-month treatment in reducing thrombotic events regardless of the D-dimer levels after 3 months of anticoagulation therapy. In addition, there was no significant increase in the risk of major bleeding in the 12-month edoxaban group relative to the 3-month edoxaban group regardless of the D-dimer levels at 3 months.

Conflict-of-interest disclosure: Y.Y. has received lecture fees from Bayer Healthcare, Bristol Myers Squibb, Pfizer, and Daiichi Sankyo, and grant support from 10.13039/501100000801Bayer Healthcare and 10.13039/501100022274Daiichi Sankyo. T.M. has received lecture fees from AstraZeneca, Bristol Myers Squibb, Daiichi Sankyo, Japan Lifeline, Kowa, Pfizer, and Tsumura; manuscript fees from Bristol Myers Squibb and Pfizer; and advisory board fees from Novartis and Teijin. Y.N. has received lecture fees from Bayer Healthcare, Bristol Myers Squibb, Pfizer, and Daiichi Sankyo. Y.O. has received lecture fees from Bayer Healthcare, Bristol Myers Squibb, Pfizer, and Daiichi Sankyo, and research funds from 10.13039/501100000801Bayer Healthcare and 10.13039/501100022274Daiichi Sankyo. N.I. has received lecture fees from Bayer Healthcare, Bristol Myers Squibb, and Daiichi Sankyo. Y.T. has received lecture fees from AstraZeneca, Chugai Pharmaceutical, Bristol Myers Squibb, Kyowa Kirin, Pfizer, Taiho Pharmaceutical, Takeda Pharmaceutical, and Daiichi Sankyo, and grant support from 10.13039/501100022274Daiichi Sankyo, 10.13039/100004325AstraZeneca, and 10.13039/501100013170Ono Pharmaceutical. S.I. has received lecture fees from Bayer Healthcare, Bristol Myers Squibb, and Daiichi Sankyo. The remaining authors declare no competing financial interests.

A complete list of the ONCO DVT Study Investigators appears in the supplemental Appendix.

## Supplementary Material

Supplemental Appendices, Tables, and Figures
