# Supplementary material for: D-dimer after 3 months of anticoagulation therapy and outcomes in cancer-associated isolated distal deep vein thrombosis
Source: Blood Vessel Thromb Hemost. 2025 Feb 24;2(2):100063. doi: 10.1016/j.bvth.2025.100063 (PMC12320435; doi:10.1016/j.bvth.2025.100063)
Supplement: Supplemental Appendices, Tables, and Figures [file BVTH_VTH-2024-000236-mmc1.pdf]

## Supplemental Material

**Supplemental Appendix**

**Supplemental Appendix 1: Study Organization**

**Principal Investigator:** Yugo YAMASHITA, Department of Cardiovascular Medicine, Graduate School of Medicine and Faculty of Medicine Kyoto University

**Protocol Committee:** Takeshi KIMURA (Co-Principal Investigators), Department of Cardiovascular Medicine, Graduate School of Medicine and Faculty of Medicine Kyoto University; Makoto MO, Department of Cardiovascular Surgery, Yokohama Minami Kyousai Hospital; Hisashi GOTO, Department of General Surgery/Department of Cardiovascular Medicine, Tohoku University; Daisuke SUETA, Department of Cardiovascular Medicine, Kumamoto University; Yugo YAMASHITA, Department of Cardiovascular Medicine, Graduate School of Medicine and Faculty of Medicine Kyoto University

**Steering Committee:** Norikazu YAMADA, Department of Cardiovascular Medicine, Kuwana City Medical Center; Taro SHIGA, Department of Cardiovascular Medicine, Cancer Institute Hospital; Tsuyoshi YAMAMOTO, Department of Cardiovascular Intensive Care, Nippon Medical School Hospital; Masafumi FUJITA, Department of Onco-Cardiology, Osaka International Cancer Institute; Kazunori OTSUI, Department of General Internal Medicine, Kobe University Hospital; Kenichi TSUJITA, Department of Cardiovascular Medicine, Kumamoto University; Satoshi IKEDA, Department of Cardiovascular Medicine, Nagasaki University; Norimichi KOITABASHI, Department of Cardiovascular Medicine, Gunma University; Shinji HISATAKE, Department of Cardiovascular Medicine, Toho University; Naohiko NAKANISHI, Department of Cardiovascular Medicine, Kyoto Prefectural University of Medicine; Kentaro JUJO, Department of Cardiovascular Medicine, Tokyo Women's Medical University; Koichiro SUGIMURA, Department of General Surgery/Department of Cardiovascular Medicine, Tohoku University; Ryoji TAKEDA, Department of Vascular Surgery, Rakuwakai Otowa Hospital; Reo HATA, Department of Cardiovascular Medicine, Kurashiki Central Hospital; Kazushige KADOTA, Department of Cardiovascular Medicine, Kurashiki Central Hospital; Toru TAKASE, Department of Cardiovascular Medicine, Kindai University Hospital; Shunichi MIYAZAKI, Department of Cardiovascular Medicine, Kindai University Hospital; Seiichi HIRAMORI, Department of Cardiovascular Medicine, Kokura Memorial Hospital; Kenji ANDO, Department of Cardiovascular Medicine, Kokura Memorial Hospital; Kite KIM, Department of Cardiovascular Medicine, Kobe City Medical Center General Hospital; Yutaka FURUKAWA, Department of Cardiovascular Medicine, Kobe City Medical Center General Hospital; Jiro SAKAMOTO, Department of Cardiovascular Medicine, Tenri Hospital; Masaharu AKAO, Department of Cardiovascular Medicine, NHO Kyoto Medical Center

**Research Operations Staff:** Yusuke YOSHIKAWA, Department of Cardiovascular Medicine, Graduate School of Medicine and Faculty of Medicine Kyoto University

**Clinical Events Committee:** Yasuhiro HAMATANI, Department of Cardiovascular Medicine, NHO Kyoto Medical Center; Kensuke TAKABAYASHI, Department of Cardiovascular Medicine, Hirakata Kohsai Hospital; Yuji NISHIMOTO, Department of Cardiovascular Medicine, Hyogo Prefectural Amagasaki General Medical Center; Yukiko NAKANO, Department of Cardiovascular Medicine, Graduate School of Medicine and Faculty of Medicine Kyoto University

**Data Safety Monitoring Committee:** Mitsuru ABE Department of Cardiovascular Medicine, NHO Kyoto Medical Center; Hidenori YAKU, Department of Cardiovascular Medicine, Mitsubishi Kyoto Hospital

**Clinical Research Organization:** MID, Inc. and Department of Cardiovascular Medicine, Graduate School of Medicine and Faculty of Medicine Kyoto University

**Monitoring officers:** Yasuaki TAKEJI, Department of Cardiovascular Medicine, Kyoto University Hospital; Yusuke YOSHIKAWA, Department of Cardiovascular Medicine, Kyoto University Hospital

**Auditors:** Chikashi TAKEDA, Department of Pharmacoepidemiology, Kyoto University Graduate School of Medicine and Public Health; Aki KUWAUCHI, Department of Pharmacoepidemiology, Kyoto University Graduate School of Medicine and Public Health

**Principal Statistician:** Takeshi MORIMOTO, Department of Clinical Epidemiology, Hyogo College of Medicine

**Supplemental Appendix 2: Participating Centers**

Department of Cardiovascular Medicine, Kyoto University Hospital (Yugo YAMASHITA), Department of Onco-Cardiology, Osaka International Cancer Institute (Masafumi FUJITA), Department of Cardiovascular Medicine, Saiseikai Noe Hospital (Ichiro KOUCHI), Department of Cardiology, Osaka Red Cross Hospital (Tsukasa INADA), Department of Cardiovascular Medicine, Japanese Red Cross Otsu Hospital (Kazuaki KAITANI), Department of Cardiovascular Medicine, Kakogawa Central City Clinics (Hiroaki NAKAMURA), Department of Cardiovascular Medicine, Cancer Institute Hospital (Taro SHIGA), Department of Vascular Surgery, Kansai Medical University Medical Center (Nobuko YAMAMOTO), Department of Cardiovascular Medicine, University Hospital Kyoto Prefectural University of Medicine (Satoaki MATOBA), Department of Cardiovascular Surgery, Kyorin University Faculty of Medicine (Yutaka HOSOI), Department of Cardiovascular Medicine, Kindai University Hospital (Gaku NAKAZAWA), Department of Cardiovascular Medicine, Kumamoto University Hospital (Daisuke SUETA), Department of Cardiovascular Medicine, Kurashiki Central Hospital (Kazushige KADOTA), Department of Cardiovascular Surgery, Kurume University Hospital (Shinichi HIROMATSU), Department of Cardiovascular Medicine, Kuwana City Medical Center (Norikazu YAMADA), Department of Cardiovascular Medicine, Gunma University (Norimichi KOITABASHI), Department of Cardiovascular Medicine, Kobe City Medical Center General Hospital (Yutaka FURUKAWA), Department of General Internal Medicine, Kobe University Hospital (Kazunori OTSUI), Department of Cardiovascular Medicine, Kohka Public Hospital (Tomohiro DOUKA), Department of Cardiovascular Surgery, Fukushima Medical University Hospital (Daiki WAKAMATSU), Department of Cardiovascular Medicine, Kokura Memorial Hospital (Kenji ANDO), Department of General Internal Medicine / Department of Cardiovascular Medicine, National Cancer Center Hospital (Masaaki SHOJI), Department of Cardiovascular Medicine, NHO Okayama Medical Center (Hiroto SHIMOKAWAHARA), Department of Cardiovascular Medicine, NHO Kyoto Medical Center (Kosuke DOI), Department of Cardiovascular Medicine, Saiseikai Yokohamashi Nanbu Hospital (Tsutomu ENDO), Department of Cardiovascular Surgery, Saiseikai Wakayama Hospital (Atsutoshi HATADA), Department of Cardiovascular Medicine, Saku Central Hospital Advanced Care Center (Yoshikazu YAZAKI), Department of Cardiovascular Medicine, Shiga General Hospital (Takeshi UENO), Department of Cardiovascular Medicine, Shizuoka Cancer Center (Nao MURAOKA), Department of Cardiovascular Medicine, Shizuoka City Shizuoka Hospital (Ryuzo NAWATA), Department of Respiratory Medicine and Clinical Oncology, Shimane University Hospital (Yukari TSUBATA), Department of Cardiovascular Medicine, Shimada General Medical Center (Yoshiaki TSUYUKI), Department of Cardiology, St. Marianna University School of Medicine (Yasuhiro TANABE), Department of Cardiovascular Medicine, Medical Research Institute Kitano Hospital (Moriaki INOKO), Department of Obstetrics and Gynecology, University of Tsukuba Hospital (Toyomi SATO), Department of Cardiovascular Medicine, Tenri Hospital (Toshihiro TAMURA), Department of Cardiovascular Medicine, Tokyo Women’s Medical University Hospital (Yuichiro

MINAMI), Department of Cardiovascular Medicine, Tokyo Metropolitan Tama Medical Center (Hiroyuki TANAKA), Department of Cardiovascular Medicine, Toho University Ohashi Medical Center (Nobutaka IKEDA), Department of Cardiovascular Medicine, Toho University Omori Medical Center (Shinji HISATAKE), Department of General Surgery, Tohoku University Hospital (Hisashi GOTO), Department of Cardiovascular Medicine, Nagasaki University Hospital (Koji MAEMURA), Department of Obstetrics and Gynecology, Nara Medical University Hospital (Ryuji KAWAGUCHI), Department of Cardiovascular Intensive Care, Nippon Medical School Hospital (Tsuyoshi YAMAMOTO), Department of Cardiovascular Medicine, Japanese Red Cross Wakayama Medical Center (Shojiro TATSUSHIMA), Department of Cardiovascular Medicine, Hyogo Prefectural Amagasaki General Medical Center (Yukihiro SATO), Department of Cardiovascular Medicine, Hirakata Kohsai Hospital (Shoji KITAGUCHI), Department of Cardiovascular Medicine, Fukui Prefectural Hospital (Susumu FUJINO), Department of Vascular Surgery, Saiseikai Yahata General Hospital (Shinsuke MII), Department of Cardiovascular Medicine, Fujisawa City Hospital (Kengo TSUKAHARA), Department of Cardiovascular Medicine, Makiminato Central Hospital (Naoya MAEHIRA), Department of Cardiovascular Medicine, Mie University Hospital (Kaoru DOHI), Department of Cardiovascular Medicine, Mitsubishi Kyoto Hospital (Takafumi YOKOMATSU), Department of Cardiovascular Medicine, Japanese Red Cross Musashino Hospital (Takashi ASHIKAGA), Department of Cardiovascular Surgery, Yokohama Minami Kyousai Hospital (Makoto MO), Hospital Department of Cardiovascular Medicine, Yokohama Rosai Hospital (Kazuhiko YUMOTO), Department of Vascular Surgery, Rakuwakai Otowa Hospital (Ryoji TAKEDA), Department of Cardiovascular Medicine, Niigata University Graduate School of Medicine and Dentistry (Shinya FUJIKI), Department of Internal Medicine, Niigata Cancer Center Niigata Hospital (Yuji OKURA), Department of Surgery of the Lower Gastrointestinal Surgery, Hyogo College of Medicine (Jihyung SONG)

**Supplemental Appendix 3: Definition of the baseline characteristics**

Diabetes: Blood glucose level ≥200 mg/dl at 2 or more hours after loading in a glucose tolerance test, casual blood glucose ≥200 mg/dl, fasting blood glucose ≥126 mg/dl, or hemoglobin A1c ≥6.5%. Even when the above tests are not performed, diabetes is defined if the patient has already been clinically diagnosed with diabetes or is taking antidiabetes medication. Heart failure: Cases that satisfy any of the following criteria are defined as heart failure: history of a hospitalization for heart failure, clinical heart failure symptoms at a New York Heart Association II (can walk on flat ground but cannot jog) or higher, and left ventricular ejection fraction <40%. History of major bleeding: Cases that satisfy any of the following criteria are defined as having a history of major bleeding: a history of bleeding into vital organs; history of bleeding that required a blood transfusion; history of bleeding with a reduction in the hemoglobin of ≥2 g/dl; or a history of bleeding that required fluid transfusion, vasopressors, or surgical treatment. Transient risk factors for venous thromboembolism included recent surgery, recent immobilization, long-distance travel, central venous catheter use, pregnancy or puerperium, recent leg trauma, fracture or burn, severe infection, and estrogen use. Anemia is diagnosed if the hemoglobin level was <13 g/dL for men and <12 g/dL for women. Eastern Cooperative Oncology Group (ECOG) performance status (PS): 0, fully active, at pre-disease performance levels without restriction. (1) Restricted physically strenuous activity but ambulatory and able to carry out work of a light and sedentary nature. (2) Ambulatory and capable of all self-care but unable to carry out any work activities. Up and about more than 50% of waking hours. (3) Capable of limited self-care only, confined to bed or chair more than 50% of waking hours. (4) Completely disabled. Cannot carry on any self-care. Totally confined to bed or a chair.

## Supplemental Appendix 4: Definition of the endpoints

### Asymptomatic recurrent VTE

New or worsening thrombus images in any imaging tests during follow-up without any symptoms.

The appearance of new or worsening thrombus images in the pulmonary arteries and deep veins on imaging tests (ultrasonography of lower limb vein system, CT examination, pulmonary perfusion scintigraphy, pulmonary angiography, venography) that do not match the definition of a symptomatic VTE recurrence and are not associated with new or worsening symptoms.

### Clinically relevant non-major bleeding (major and non-major bleeding events)

Major bleeding is defined as symptoms indicating bleeding and applicable to any of the following:

1) Fatal bleeding
2) Symptomatic bleeding in a critical area or organ (intracranial, intraspinal, intraocular, retroperitoneal, intraarticular or pericardial, or intramuscular with compartment syndrome)
3) Bleeding causing a decrease of ≥2 g/dL in the hemoglobin level or leading to a transfusion of ≥2 units of whole blood or red cells.

Clinically relevant non-major bleeding is defined as a clinically overt bleed (including bleeds detected only with imaging diagnostics) that does not meet the criteria for a major bleed but leads to at least one of the following:

1) A physician-guided medical intervention
2) A hospital admission or further treatment for bleeding
3) Face-to-face medical examination by physician (not simply telephone or electronic communication)

**Supplemental Tables**

**Supplemental Table 1. Comparison of patient characteristics**

| | Low D-dimer subgroup (D-dimer <1.0 μg/mL at 3 months) (N=308) | | | | High D-dimer subgroup (D-dimer ≥1.0 μg/mL at 3 months) (N=211) | | | |
|---|---|---|---|---|---|---|---|---|
| | 12-month edoxaban group (N=143) | | 3-month edoxaban group (N=165) | | 12-month edoxaban group (N=108) | | 3-month edoxaban group (N=103) | |
| **Baseline characteristics** | | | | | | | | |
| Age, years | 70.7 | ±9.4 | 68.9 | ±10.7 | 71.3 | ±9.6 | 70.7 | ±10.1 |
| Age ≥75 years | 57 | (40) | 58 | (35) | 45 | (42) | 38 | (37) |
| Male sex | 37 | (26) | 33 | (20) | 40 | (37) | 28 | (27) |
| Body weight, kg | 57.7 | ±13.0 | 55.5 | ±12.9 | 56.4 | ±11.1 | 53.8 | ±10.4 |
| Body mass index, $kg/m^2$ | 23.3 | ±4.3 | 22.7 | ±4.5 | 22.6 | ±3.9 | 21.9 | ±3.70 |
| Symptoms at baseline | 23 | (16) | 32 | (19) | 24 | (22) | 23 | (22) |
| Site of thrombosis | | | | | | | | |
| Bilateral | 54 | (38) | 49 | (30) | 46 | (43) | 40 | (39) |
| Right side | 44 | (31) | 46 | (28) | 17 | (16) | 30 | (29) |
| Left side | 45 | (31) | 70 | (42) | 45 | (42) | 33 | (32) |
| Standard dose of edoxaban (60 mg per day)* | 41 | (29) | 42 | (25) | 31 | (29) | 22 | (21) |
| Lower dose of edoxaban (30 mg per day) * | 102 | (71) | 123 | (75) | 77 | (71) | 81 | (79) |
| **Cancer status** | | | | | | | | |
| Newly diagnosed cancer within 6 months | 92 | (64) | 115 | (70) | 61 | (56) | 72 | (70) |
| Chemotherapy performed within 6 months | 67 | (47) | 77 | (47) | 61 | (56) | 52 | (50) |

| | | | | | | | | |
|---|---|---|---|---|---|---|---|---|
| Radiotherapy performed within 6 months | 9 | (6) | 13 | (8) | 9 | (8) | 14 | (14) |
| Recurrent cancer | 14 | (10) | 20 | (12) | 11 | (10) | 6 | (6) |
| Metastatic disease | 21 | (15) | 33 | (20) | 30 | (28) | 34 | (33) |
| ECOG performance status | | | | | | | | |
| 0 | 89 | (62) | 96 | (58) | 56 | (52) | 40 | (39) |
| 1 | 40 | (28) | 55 | (33) | 26 | (24) | 42 | (41) |
| ≥2 | 14 | (10) | 14 | (9) | 26 | (24) | 21 | (20) |
| **Comorbidities** | | | | | | | | |
| Hypertension | 58 | (41) | 65 | (39) | 49 | (45) | 48 | (47) |
| Diabetes | 24 | (17) | 20 | (12) | 22 | (20) | 18 | (17) |
| Heart failure | 1 | (1) | 1 | (1) | 3 | (3) | 0 | (0) |
| History of stroke | 8 | (6) | 6 | (4) | 3 | (3) | 6 | (6) |
| History of VTE | 7 | (5) | 7 | (4) | 11 | (10) | 4 | (4) |
| History of major bleeding | 2 | (1) | 9 | (5) | 4 | (4) | 5 | (5) |
| Transient risk factors for VTE | 34 | (24) | 41 | (25) | 30 | (28) | 20 | (19) |
| Recent surgery within 2 months | 68 | (48) | 75 | (45) | 44 | (41) | 33 | (32) |
| **Laboratory values at diagnosis** | | | | | | | | |
| Creatinine clearance ≤50 ml/min | 29 | (20) | 28 | (17) | 24 | (22) | 22 | (21) |
| Anemia)† | 77 | (54) | 106 | (64) | 87 | (81) | 71 | (69) |
| Platelet count, /μL | 24.7 | ±10.3 | 25.4 | ±11.7 | 24.9 | ±11.0 | 26.1 | ±12.9 |
| D-dimer at randomization, μg/mL | 3.9 | (1.7-8.7) | 3.9 | (2.1-8.8) | 8.4 | (3.5-15.2) | 6.2 | (2.5-13.6) |
| D-dimer at 3 months, μg/mL | 0.5 | (0.5-0.7) | 0.5 | (0.5-0.7) | 2.5 | (1.4-4.9) | 2.4 | (1.5-6.4) |
| **Concomitant medication** | | | | . | | | | |

| | | | | | | | | |
|---|---|---|---|---|---|---|---|---|
| Antiplatelet | 12 | (8) | 11 | (7) | 5 | (5) | 7 | (7) |
| Steroid | 12 | (8) | 18 | (11) | 17 | (16) | 19 | (18) |
| Statin | 37 | (26) | 33 | (20) | 21 | (19) | 19 | (18) |
| Proton pump inhibitor | 44 | (31) | 45 | (27) | 40 | (37) | 40 | (39) |
| $H_2$ blocker | 7 | (5) | 12 | (7) | 0 | (0) | 10 | (10) |

Ccontinuous variables are presented as the medians (interquartile ranges) or means ± standard deviations. Categorical values are presented as numbers (%).

*Edoxaban is administered at a lower dose of 30 mg/day for the participants with a creatinine clearance of 30–50 mL/min, body weight of ≤ 60 kg, or for those with concomitant treatment with potent P-glycoprotein inhibitors.

†Anemia is defined as a hemoglobin level of < 13 g/dL for men and < 12 g/dL for women.

ECOG, Eastern Cooperative Oncology Group; VTE, venous thromboembolism; $H_2$, histamine

**Supplemental Table 2. Types of cancers**

| N (%) | Low D-dimer subgroup (N=308) | | High D-dimer subgroup (N=211) | |
|---|---|---|---|---|
| | 12-month edoxaban group (N=143) | 3-month edoxaban group (N=165) | 12-month edoxaban group (N=108) | 3-month edoxaban group (N=103) |
| Lung | 13 (9) | 12 (7) | 15 (14) | 18 (17) |
| Colon | 14 (10) | 18 (11) | 8 (7) | 10 (10) |
| Stomach | 6 (4) | 11 (7) | 6 (5) | 5 (5) |
| Uterus | 20 (14) | 25 (15) | 17 (16) | 14 (13) |
| Blood | 7 (5) | 12 (8) | 5 (4) | 3 (3) |
| Ovary | 24 (17) | 32 (19) | 13 (12) | 11 (11) |
| Prostate | 1 (1) | 3 (2) | 5 (4) | 0 (0) |
| Breast | 14 (10) | 10 (6) | 7 (6) | 1 (1) |
| Pancreas | 12 (8) | 6 (4) | 8 (7) | 17 (16) |
| Bladder | 7 (5) | 5 (3) | 4 (4) | 7 (7) |
| Brain | 3 (2) | 1 (1) | 3 (3) | 2 (2) |
| Kidney/ureter | 2 (1) | 2 (1) | 2 (2) | 4 (4) |
| Esophagus | 3 (2) | 4 (2) | 3 (3) | 0 (0) |
| Gall bladder/bile duct | 3 (2) | 2 (1) | 1 (1) | 0 (0) |
| Skin | 1 (1) | 5 (3) | 1 (1) | 0 (0) |
| Thyroid gland | 0 (0) | 0 (0) | 1 (1) | 0 (0) |
| Liver | 0 (0) | 4 (2) | 2 (2) | 0 (0) |
| Multiple | 2 (1) | 2 (1) | 4 (4) | 2 (2) |
| Others | 11 (8) | 11 (7) | 3 (3) | 9 (9) |

**Supplemental Table 3. Reasons for persistent edoxaban discontinuation**

| N (%) | Low D-dimer subgroup | | High D-dimer subgroup | |
|---|---|---|---|---|
| | **12-month edoxaban group (N=36)** | **3-month edoxaban group (N=158)** | **12-month edoxaban group (N=53)** | **3-month edoxaban group (N=93)** |
| Per-protocol discontinuation | 0 (0) | 147 (93) | 0 (0) | 57 (62) |
| Due to drug side effect | 0 (0) | 0 (0) | 9 (17) | 4 (4) |
| Due to bleeding events | 9 (25) | 1 (1) | 13 (25) | 14 (15) |
| Due to operation | 3 (8) | 2 (1) | 5 (9) | 2 (2) |
| Patient's decision | 5 (14) | 0 (0) | 5 (9) | 3 (3) |
| Due to cancer progression | 6 (17) | 0 (0) | 16 (30) | 3 (3) |
| Physician's decision | 4 (11) | 0 (0) | 2 (4) | 0 (0) |
| Others | 9 (25) | 8 (5) | 3 (6) | 10 (11) |

## Supplemental Figures

### Asymptomatic recurrent VTE

A Low D-dimer subgroup

B High D-dimer subgroup

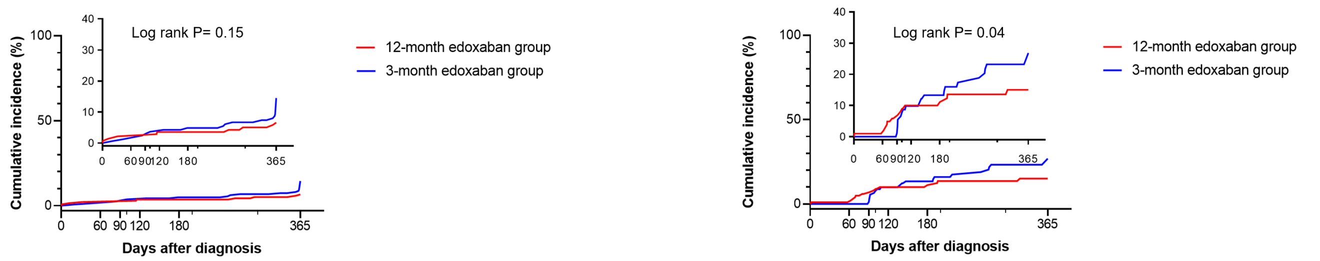


**A**

| | 0 day | 60 days | 90 days | 120 days | 180 days | 365 days |
|---|---|---|---|---|---|---|
| 12-month edoxaban group | | | | | | |
| N of patients with event | | 3 | 3 | 5 | 5 | 9 |
| N of patients at risk | 143 | 140 | 140 | 138 | 136 | 124 |
| Cumulative incidence (%) | | 2.1 | 2.1 | 3.5 | 3.5 | 6.5 |
| 3-month edoxaban group | | | | | | |
| N of patients with event | | 2 | 4 | 6 | 8 | 23 |
| N of patients at risk | 165 | 163 | 161 | 159 | 156 | 140 |
| Cumulative incidence (%) | | 1.2 | 2.4 | 3.6 | 4.9 | 14.4 |

**B**

| | 0 day | 60 days | 90 days | 120 days | 180 days | 365 days |
|---|---|---|---|---|---|---|
| 12-month edoxaban group | | | | | | |
| N of patients with event | | 1 | 6 | 10 | 11 | 14 |
| N of patients at risk | 108 | 101 | 92 | 86 | 75 | 57 |
| Cumulative incidence (%) | | 0.9 | 5.9 | 10.0 | 11.1 | 18.3 |
| 3-month edoxaban group | | | | | | |
| N of patients with event | | 0 | 1 | 9 | 12 | 21 |
| N of patients at risk | 103 | 96 | 92 | 80 | 69 | 42 |
| Cumulative incidence (%) | | 0 | 1.1 | 9.8 | 13.3 | 26.9 |

**Supplemental Figure 1. Kaplan–Meier curves for asymptomatic recurrent VTE comparing 12-month and 3-month edoxaban groups in the subgroups stratified by D-dimer levels at 3 months; (A) Low D-dimer subgroup and (B) High D-dimer subgroup**

VTE, venous thromboembolism.

## All clinically relevant bleeding

A

### Low D-dimer subgroup

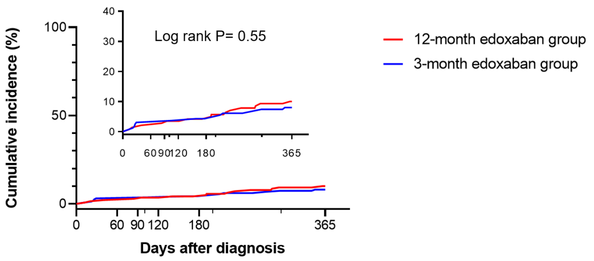


| | 0 day | 60 days | 90 days | 120 days | 180 days | 365 days |
|---|---|---|---|---|---|---|
| 12-month edoxaban group | | | | | | |
| N of patients with event | | 3 | 4 | 5 | 6 | 14 |
| N of patients at risk | 143 | 140 | 139 | 138 | 135 | 119 |
| Cumulative incidence (%) | | 2.1 | 2.8 | 3.5 | 4.2 | 10.0 |
| 3-month edoxaban group | | | | | | |
| N of patients with event | | 5 | 5 | 6 | 7 | 13 |
| N of patients at risk | 165 | 160 | 160 | 159 | 156 | 142 |
| Cumulative incidence (%) | | 3.0 | 3.0 | 3.6 | 4.2 | 8.0 |

B

### High D-dimer subgroup

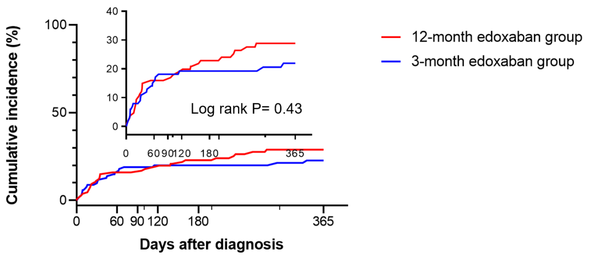


| | 0 day | 60 days | 90 days | 120 days | 180 days | 365 days |
|---|---|---|---|---|---|---|
| 12-month edoxaban group | | | | | | |
| N of patients with event | | 17 | 17 | 20 | 24 | 27 |
| N of patients at risk | 108 | 88 | 86 | 83 | 71 | 52 |
| Cumulative incidence (%) | | 15.9 | 15.9 | 18.8 | 22.9 | 28.9 |
| 3-month edoxaban group | | | | | | |
| N of patients with event | | 16 | 19 | 20 | 20 | 22 |
| N of patients at risk | 103 | 82 | 77 | 74 | 69 | 50 |
| Cumulative incidence (%) | | 15.8 | 18.9 | 20.0 | 20.0 | 22.7 |

**Supplemental Figure 2. Kaplan–Meier curves for all clinically relevant bleeding comparing between 12-month and 3-month edoxaban groups in the subgroups stratified by D-dimer levels at 3 months. (A) Low D-dimer subgroup. (B) High D-dimer subgroup**

## All-cause death

A

### Low D-dimer subgroup

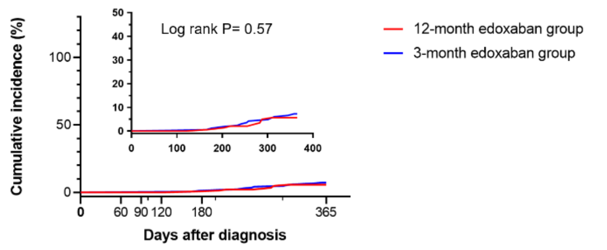


| | 0 day | 60 days | 90 days | 120 days | 180 days | 365 days |
|---|---|---|---|---|---|---|
| 12-month edoxaban group | | | | | | |
| N of patients with event | | 0 | 0 | 0 | 1 | 8 |
| N of patients at risk | 143 | 143 | 143 | 143 | 141 | 132 |
| Cumulative incidence (%) | | 0 | 0 | 0 | 0.7 | 5.7 |
| 3-month edoxaban group | | | | | | |
| N of patients with event | | 0 | 0 | 0 | 2 | 12 |
| N of patients at risk | 165 | 165 | 165 | 165 | 163 | 152 |
| Cumulative incidence (%) | | 0 | 0 | 0 | 1.2 | 7.3 |

B

### High D-dimer subgroup

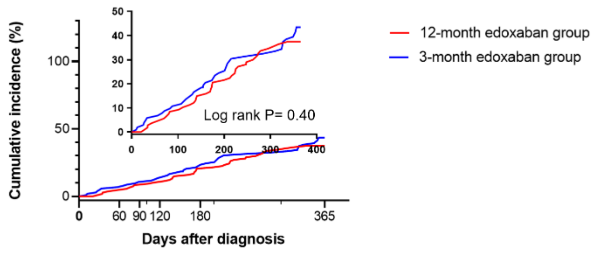


| | 0 day | 60 days | 90 days | 120 days | 180 days | 365 days |
|---|---|---|---|---|---|---|
| 12-month edoxaban group | | | | | | |
| N of patients with event | | 5 | 9 | 11 | 22 | 40 |
| N of patients at risk | 108 | 102 | 98 | 96 | 85 | 67 |
| Cumulative incidence (%) | | 4.7 | 8.4 | 10.3 | 20.6 | 37.4 |
| 3-month edoxaban group | | | | | | |
| N of patients with event | | 7 | 11 | 14 | 24 | 44 |
| N of patients at risk | 103 | 96 | 92 | 89 | 79 | 56 |
| Cumulative incidence (%) | | 6.8 | 10.7 | 13.6 | 23.5 | 43.5 |

**Supplemental Figure 3. Kaplan–Meier curves for all-cause death comparing between 12-month and 3-month edoxaban groups in the subgroups stratified by D-dimer levels at 3 months. (A) Low D-dimer subgroup. (B) High D-dimer subgroup**

## Primary endpoint

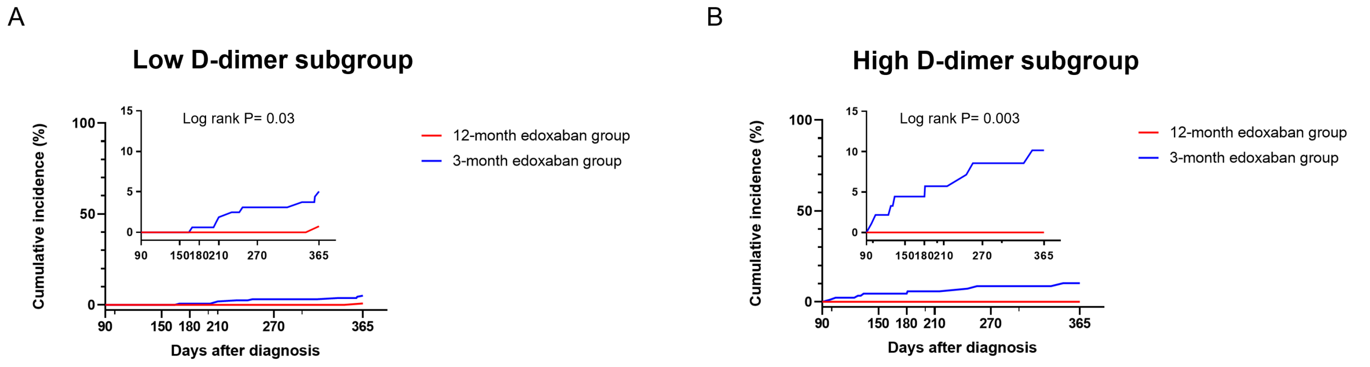


| | 90 day | 150 days | 180 days | 210 days | 270 days | 365 days |
|---|---|---|---|---|---|---|
| 12-month edoxaban group | | | | | | |
| N of patients with event | | 0 | 0 | 0 | 0 | 1 |
| N of patients at risk | 143 | 142 | 141 | 140 | 138 | 132 |
| Cumulative incidence (%) | | 0 | 0 | 0 | 0 | 0.8 |
| 3-month edoxaban group | | | | | | |
| N of patients with event | | 0 | 1 | 3 | 5 | 8 |
| N of patients at risk | 164 | 164 | 162 | 160 | 153 | 145 |
| Cumulative incidence (%) | | 0 | 0.6 | 1.9 | 3.1 | 5.1 |

| | 90 day | 150 days | 180 days | 210 days | 270 days | 365 days |
|---|---|---|---|---|---|---|
| 12-month edoxaban group | | | | | | |
| N of patients with event | | 0 | 0 | 0 | 0 | 0 |
| N of patients at risk | 97 | 90 | 84 | 83 | 73 | 66 |
| Cumulative incidence (%) | | 0 | 0 | 0 | 0 | 0 |
| 3-month edoxaban group | | | | | | |
| N of patients with event | | 4 | 4 | 5 | 7 | 8 |
| N of patients at risk | 92 | 81 | 76 | 70 | 64 | 51 |
| Cumulative incidence (%) | | 4.5 | 4.5 | 5.7 | 8.6 | 10.2 |

**Supplemental Figure 4. Landmark analysis beyond 90 days for the primary endpoint**

The primary endpoint is a composite of symptomatic recurrent VTE or VTE-related death.

VTE, venous thromboembolism

## Major secondary endpoint

A

### Low D-dimer subgroup

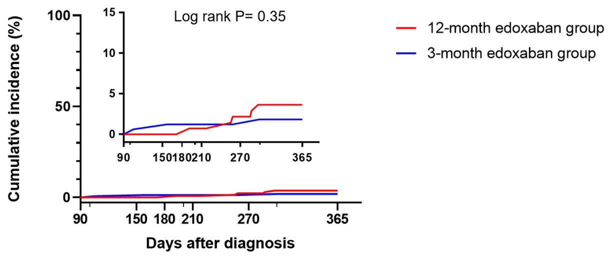


| | 90 day | 150 days | 180 days | 210 days | 270 days | 365 days |
|---|---|---|---|---|---|---|
| 12-month edoxaban group | | | | | | |
| N of patients with event | | 0 | 0 | 1 | 3 | 5 |
| N of patients at risk | 143 | 142 | 141 | 139 | 135 | 128 |
| Cumulative incidence (%) | | 0 | 0 | 0.7 | 2.2 | 3.6 |
| 3-month edoxaban group | | | | | | |
| N of patients with event | | 1 | 2 | 2 | 2 | 3 |
| N of patients at risk | 165 | 164 | 161 | 161 | 157 | 151 |
| Cumulative incidence (%) | | 0.6 | 1.2 | 1.2 | 1.2 | 1.8 |

B

### High D-dimer subgroup

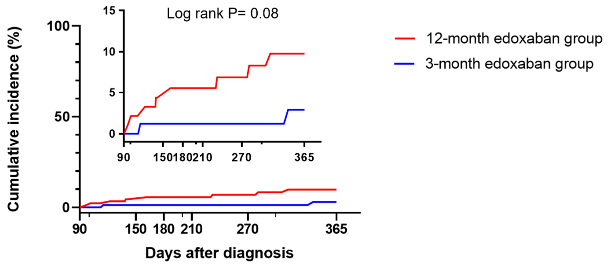


| | 90 day | 150 days | 180 days | 210 days | 270 days | 365 days |
|---|---|---|---|---|---|---|
| 12-month edoxaban group | | | | | | |
| N of patients with event | | 4 | 5 | 5 | 6 | 8 |
| N of patients at risk | 92 | 83 | 78 | 77 | 66 | 60 |
| Cumulative incidence (%) | | 4.4 | 5.6 | 5.6 | 6.9 | 9.8 |
| 3-month edoxaban group | | | | | | |
| N of patients with event | | 1 | 1 | 1 | 1 | 2 |
| N of patients at risk | 83 | 77 | 71 | 67 | 65 | 53 |
| Cumulative incidence (%) | | 1.2 | 1.2 | 1.2 | 1.2 | 2.9 |

**Supplemental Figure 5. Landmark analysis beyond 90 days for the major secondary endpoint**

The major secondary endpoint is major bleeding as defined according to the International Society on Thrombosis and Haemostasis criteria and consists of fatal bleeding, symptomatic bleeding in a critical area or organ, and bleeding causing a reduction in the hemoglobin levels ≥ 2 g/dL or leading to a transfusion of ≥ 2 units of whole blood or red cells.

## Primary endpoint

**A**

### Low D-dimer subgroup

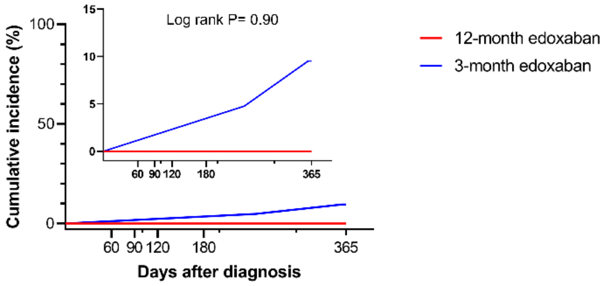


| | 0 day | 60 days | 90 days | 120 days | 180 days | 365 days |
|---|---|---|---|---|---|---|
| 12-month edoxaban group | | | | | | |
| N of patients with event | | 0 | 0 | 0 | 0 | 0 |
| N of patients at risk | 30 | 30 | 30 | 30 | 30 | 29 |
| Cumulative incidence (%) | | 0 | 0 | 0 | 0 | 0 |
| 3-month edoxaban group | | | | | | |
| N of patients with event | | 0 | 0 | 0 | 0 | 2 |
| N of patients at risk | 21 | 21 | 21 | 21 | 21 | 19 |
| Cumulative incidence (%) | | 0 | 0 | 0 | 0 | 9.5 |

**B**

### High D-dimer subgroup

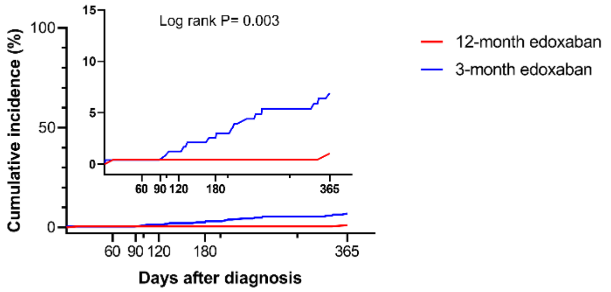


| | 0 day | 60 days | 90 days | 120 days | 180 days | 365 days |
|---|---|---|---|---|---|---|
| 12-month edoxaban group | | | | | | |
| N of patients with event | | 1 | 1 | 1 | 1 | 2 |
| N of patients at risk | 221 | 214 | 210 | 208 | 195 | 169 |
| Cumulative incidence (%) | | 0.5 | 0.5 | 0.5 | 0.5 | 1.0 |
| 3-month edoxaban group | | | | | | |
| N of patients with event | | 1 | 1 | 3 | 6 | 15 |
| N of patients at risk | 247 | 239 | 235 | 231 | 217 | 177 |
| Cumulative incidence (%) | | 0.4 | 0.4 | 1.3 | 2.6 | 6.9 |

**Supplemental Figure 6. Sensitivity analysis with the cut-off value of D-dimer as 0.5 µg/mL for the primary endpoint.**

The primary endpoint is a composite of symptomatic recurrent VTE or VTE-related death.

VTE, venous thromboembolism

## Major secondary endpoint

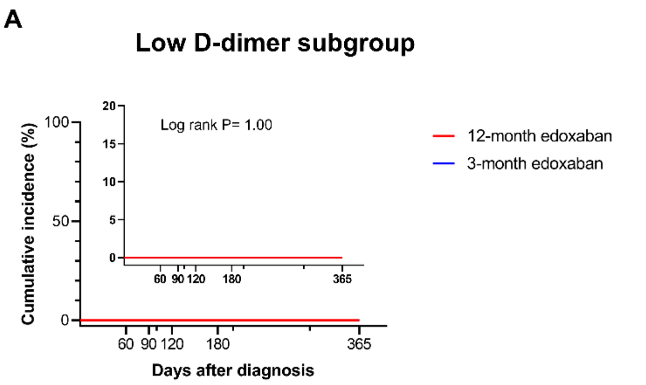


| | 0 day | 60 days | 90 days | 120 days | 180 days | 365 days |
|---|---|---|---|---|---|---|
| 12-month edoxaban group | | | | | | |
| N of patients with event | | 0 | 0 | 0 | 0 | 0 |
| N of patients at risk | 30 | 30 | 30 | 30 | 30 | 29 |
| Cumulative incidence (%) | | 0 | 0 | 0 | 0 | 0 |
| 3-month edoxaban group | | | | | | |
| N of patients with event | | 0 | 0 | 0 | 0 | 0 |
| N of patients at risk | 21 | 21 | 21 | 21 | 21 | 21 |
| Cumulative incidence (%) | | 0 | 0 | 0 | 0 | 0 |

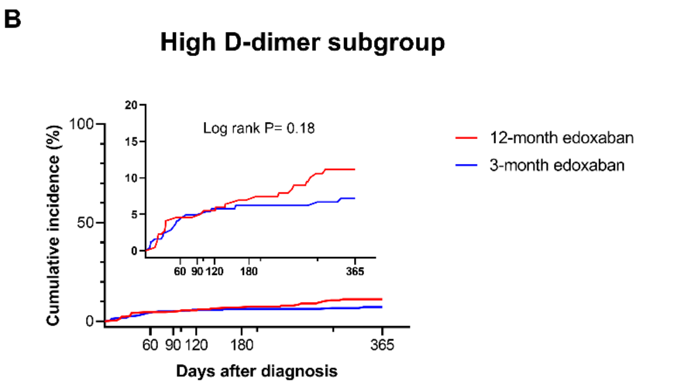


| | 0 day | 60 days | 90 days | 120 days | 180 days | 365 days |
|---|---|---|---|---|---|---|
| 12-month edoxaban group | | | | | | |
| N of patients with event | | 10 | 10 | 12 | 15 | 23 |
| N of patients at risk | 221 | 207 | 205 | 202 | 189 | 159 |
| Cumulative incidence (%) | | 4.6 | 4.6 | 5.5 | 6.9 | 11.1 |
| 3-month edoxaban group | | | | | | |
| N of patients with event | | 10 | 12 | 14 | 15 | 17 |
| N of patients at risk | 247 | 231 | 227 | 223 | 213 | 183 |
| Cumulative incidence (%) | | 4.1 | 4.9 | 5.8 | 6.2 | 7.2 |

**Supplemental Figure 7. Sensitivity analysis with the cut-off value of D-dimer as 0.5 μg/mL for the secondary primary endpoint.**

The major secondary endpoint is major bleeding as defined according to the International Society on Thrombosis and Haemostasis criteria and consists of fatal bleeding, symptomatic bleeding in a critical area or organ, and bleeding causing a reduction in the hemoglobin levels ≥ 2 g/dL or leading to a transfusion of ≥ 2 units of whole blood or red cells.
